# Polyaniline Nanofiber Wrapped Fabric for High Performance Flexible Pressure Sensors

**DOI:** 10.3390/polym11071120

**Published:** 2019-07-02

**Authors:** Kangning Liu, Ziqiang Zhou, Xingwu Yan, Xiang Meng, Hua Tang, Konggang Qu, Yuanyuan Gao, Ying Li, Junsheng Yu, Lu Li

**Affiliations:** 1 Research Institute for New Materials Technology, Chongqing University of Arts and Sciences, Chongqing 402160, China; 2 State Key Laboratory of Electronic Thin Films and Integrated Devices, School of Optoelectronic Science and Engineering, University of Electronic Science and Technology of China (UESTC), Chengdu 610054, China; 3 School of Chemistry and Chemical Engineering, Liaocheng University, Liaocheng 252059, China

**Keywords:** polyaniline, flexible pressure sensors, smart textile, wearable electronics

## Abstract

The rational design of high-performance flexible pressure sensors with both high sensitivity and wide linear range attracts great attention because of their potential applications in wearable electronics and human-machine interfaces. Here, polyaniline nanofiber wrapped nonwoven fabric was used as the active material to construct high performance, flexible, all fabric pressure sensors with a bottom interdigitated textile electrode. Due to the unique hierarchical structures, large surface roughness of the polyaniline coated fabric and high conductivity of the interdigitated textile electrodes, the obtained pressure sensor shows superior performance, including ultrahigh sensitivity of 46.48 kPa^−1^ in a wide linear range (<4.5 kPa), rapid response/relaxation time (7/16 ms) and low detection limit (0.46 Pa). Based on these merits, the practical applications in monitoring human physiological signals and detecting spatial distribution of subtle pressure are demonstrated, showing its potential for health monitoring as wearable electronics.

## 1. Introduction

Flexible pressure sensors which mimic the functions of natural skin by transferring various physical deformations into electronic signals have attracted tremendous attention in the applications of wearable devices [1,2], healthcare monitoring [3,4,5] and human-machine interfaces [6,7]. In order to monitor physiological signals and human motion with explicit details and without distortion, skin-like sensors endowed with both high sensitivity and a large workable pressure range are desirable [8,9]. Furthermore, flexible pressure sensors are required to be mountable and lightweight, which can be applied in human real-time physiological sensing over a long period of time without discomfort [8,10]. To date, a variety of sensing mechanisms have been developed for flexible pressure sensors, including piezoresistivity [11,12], capacitance [13], piezoelectricity [14] and triboelectricity [15]. Piezoresistive sensors exhibit great potential and have been widely applied due to the facile fabrication process, simple read-out mechanism and superior sensitivity [16]. Recently, a number of resistive-type pressure sensors based on elastomers have been engineered with different surface microstructures such as micropyramids, microdomes [17] and epidermal microstructure [18]. These pressure sensors have been reported with high sensitivity and excellent flexibility. However, either the fabrication process can be laborious or the adopted airproof elastic substrates, such as polydimethylsiloxane (PDMS) [17,19], polyethylene terephthalate (PET) [20] and polyurethane (PU) [21,22], are unfavorable for the breathable designs and large-area application. In contrast, a textile is ideal for the substrate of wearable pressure sensors with detection purposes of healthcare when mounted on human skin if it is breathable, soft and lightweight [23,24]. Moreover, textiles with intrinsic high porous microstructure and large surface roughness are especially suitable for high-performance piezoresistive pressure sensors [10].

On the other side, the selection of conductive materials is vital for the fabrication of high-performance pressure sensors. Conductive materials such as carbon black [25,26], graphene [27,28], carbon nanotubes (CNTs) [29,30] and polyaniline (PANI) [31,32,33] have been used as the conductive layers for flexible pressure sensors. Despite the progress, the trade-off between sensitivity and linear range remains a huge challenge. For example, Luo and co-workers have reported a wearable pressure sensor based on carbon-decorated textile, which exhibits a large linear range of 0–35 kPa, but very low sensitivity of 0.585 kPa^−1^ [4]. Another work reported a resistive-type pressure sensor with a relatively high sensitivity of 3.4 kPa^−1^ using silver nanowires coated on cotton. However, the linear range was only 0–200 Pa and apparent decline in sensitivity arises for a large pressure range [34].

Herein, we report a facilely-fabricated textile-based pressure sensor with ultrahigh sensitivity as well as large linearity based on a top bridge of PANI-wrapped nonwoven fabric and screen-printed interdigitated textile electrodes. PANI was in-situ synthesized on the fibers’ surface and the silver electrodes were screen-printed. Both of the two manufacturing processes are facile, low-cost and suitable for large-area and high-volume production in the future. Nonwoven fabric has a random fiber network microstructure which endows a rough surface with Gaussian random distribution (RDS). Owing to the RDS surface of the piezoresistive materials and high conductivity of interdigitated textile electrodes, our pressure sensors maintained ultra-sensitivity of 46.48 kPa^−1^ in a wide linear range (<4.5 kPa), which is among the best results for wearable pressure sensors. Moreover, the textile sensor could achieve fast response/relaxation time (7/16 ms) and a low detection limit (0.46 Pa). Based on these remarkable detective properties, our sensors could detect wrist pulse and carotid pulse signals. In addition, a proof-of-concept pressure sensor arrays has been further demonstrated for the detection to the mapping of subtle pressure distribution.

## 2. Materials and Methods

### 2.1. Materials

Commercially available nonwoven fabric and cotton substrates. PANI and Hydrochloric acid (KESHI, Chengdu, China). Conductive silver paste (ENSON CD-03, Guangzhou, China).

### 2.2. Preparation of the Screen-Printed Fabric Electrodes

The commercially conductive silver paste was printed on the pre-cleaned cotton through a screen-printing process. After drying at 80 °C for 15 min, patterned silver electrodes on the cotton substrate with a sheet resistance of about 0.37 Ω/sq. were obtained. 

### 2.3. Fabrication of the PANI-Based Pressure Sensors

At first, hydrochloric acid was used as doped acid and ammonium persulfate (APS) was used as the oxidant. After washing the non-woven fabric with a sufficient amount of ethanol and deionized (DI) water, the cloth was dried in a constant temperature drying oven and immersed in the mixed PANI/HCl solutions of different concentrations (1 mL aniline was dissolved in 40 mL HCl solutions of 0.5 mol/L, 1 mol/L and 2 mol/L, respectively). Subsequently, after ultrasonic treatment for 10 min, 150 mg ammonium persulfate (APS) was fully dissolved in 600 mL deionized water and 250 mL APS solution was added to the PANI/HCl/Cotton mixtures at different concentrations and stirred for 30 min. After the reaction was completely static, the PANI-modified cotton was taken out, rinsed with deionized water for 5 min and dried at 70 °C for 30 min. The PANI modified fabrics were marked as 0.5 mol HCl PANI/Cotton, 1.0 mol HCl PANI/Cotton and 2.0 mol HCl PANI/Cotton, respectively. The fabric electrodes were printed using the screen-printing method, followed by wrapping the PANI/Cotton sensor with a filmy 3M™ VHB™ tape.

### 2.4. Characterization of the Device

The SEM images were obtained using a field emission SEM (GeminiSEM 300, Hallbergmoos, Germany). The current, sheet resistance and mechanical force signals were recorded using a digital source meter (Keithley2400, Beaverton, OR, USA) and an electrochemical workstation (CHI 760E, Shanghai, China) in order to observe the responses of the piezoresistive sensors against multiple stimuli. The molecular internal structure of the fabric was tested and analyzed by Fourier Transform Infrared Spectrometer (FTIR) (Shimadzu uv-3600, Kyoto, Japan). The attenuated total reflection mode was adopted and the test resolution was 0.4 cm^−1^. The information of the chemical bond or functional group in the fabric can be determined by the analysis of infrared spectrum. The 3D morphology of the PANI-coated nonwoven fabric was characterized by a laser scanning confocal microscope (OPTELICS C130, Kanagawa, Japan).

## 3. Results and Discussion

### 3.1. Device Fabrication and Characterization

**Figure 1:**
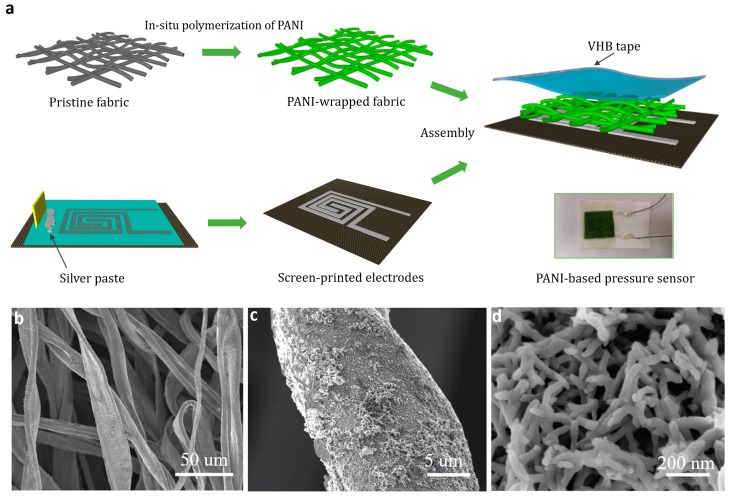
Fabrication process of the polyaniline (PANI)-coated pressure sensor. (**a**) Schematic illustration of the fabrication procedure of the flexible pressure sensor and its digital photograph; (**b**–**d**) Scanning electron microscopy (SEM) images of the PANI-coated fabric with different magnifications.

The fabrication procedure of the PANI-based pressure sensor is illustrated in Figure 1a. The flexible textile-based pressure sensors utilize PANI-modified non-woven fabric as the piezoresistive layer, which is covered in screen-printed interdigitated electrodes at the bottom. A thin VHB tape was used to fix these two layers. The digital photo of the constructed sensing device is shown in Figure 1a:

SEM was performed to observe the surface microstructure of the PANI coated conductive fabric. In Figure 1b, the fibers’ distribution of the non-woven fabric adopted by the device are irregularly aligned, which is different from the fiber of the woven fabric with a distinct warp and weft. Such a structure allows a larger air gap between the fibers. Figure 1c,d are magnified images of PANI conductive fibers, from which we observe that the cotton fiber surface is covered with a cluster network of PANI nanofibers (approximately 50 nm in diameter). The closely packed PANI nanofibers further increased the fabric surface roughness and electric conductivity.

**Figure 2:**
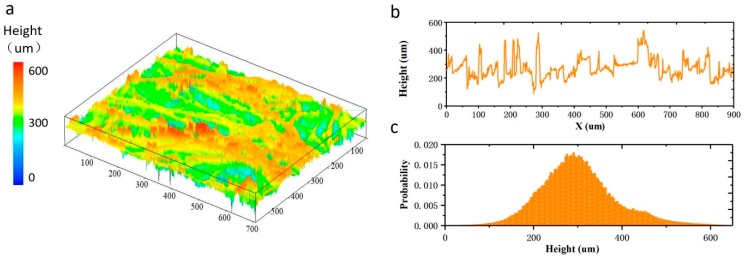
Characterization of the prepared Gaussian random distribution samples. (**a**) Three-dimensional (3D) morphology of the PANI coated fabric; (**b**) Height profile corresponding to the marked cross profile on the diagonal; (**c**) Probability distribution of the surface height.

**Figure 3:**
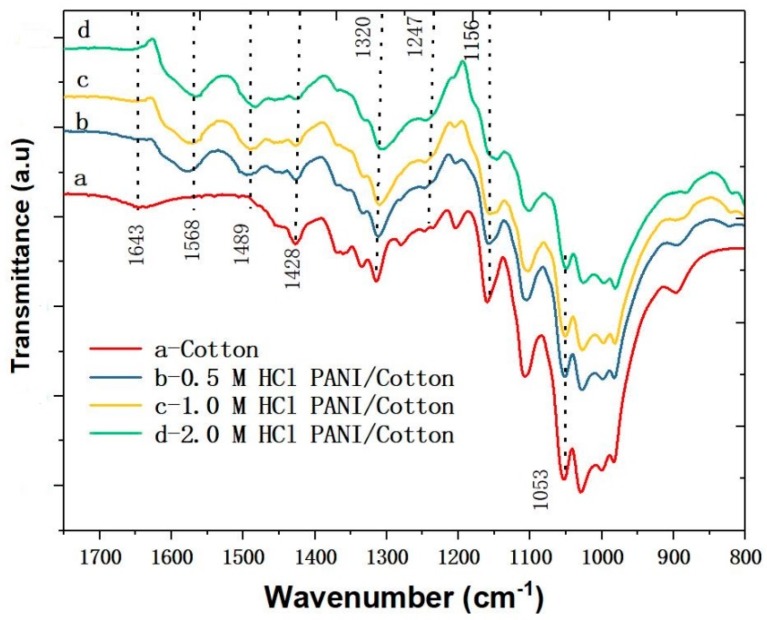
Infrared absorption spectra of the PANI-coated fabrics.

Figure 2a shows the 3D surface profile of the PANI coated conductive fabric. The conductive fabric surface has an uneven height distribution within the range of 100–500 μm (Figure 2b). In addition, Figure 2c is the probability distribution of the surface height of the fabric, indicating that the surface height of the non-woven fabric is random and close to the gaussian distribution with the center of 300 μm. Such a randomly distributed surface is conducive to increase the linearity of the device response. Spectral analysis was also performed to confirm its infrared absorption characteristics. The infrared absorption spectra of the cotton fabric and PANI coated conductive fabric were measured by FTIR, and the characteristic peaks were observed (Figure 3). The infrared absorption peaks of these samples indicate that the composite fabric has the characteristic absorption peaks of both cotton fiber and PANI. Besides, the characteristic peaks corresponding to the cellulose structure of cotton fabric are 1643 cm^−1^, 1428 cm^−1^, 1156 cm^−1^ and 1053 cm^−1^, respectively. The absorption peak belonging to 1643 cm^−1^ originated from the bending vibration of the H–O–H bond in water absorbed by the fabric. The characteristic peaks belonging to 1428 cm^−1^ were derived from the bending vibration caused by aliphatic –CH_2_ in cellulose. At 1053 cm^−1^, the high intensity is due to the presence of C–O–C pyranose ring skeleton in the cellulose which produces vibrations. Compared with the infrared spectrum of pure cotton fabric, the infrared spectrum of PANI/Cotton at 1568 cm^−1^ and 1489 cm^−1^ (Figure 3) was wide and strong, which was derived from the characteristic peaks of the benzene ring. The absorption peak at 1568 cm^−1^ represents the stretching vibration of the ketone diimide unit (C=N and C=C), which at 1489 cm^−1^ is attributed to the stretching vibration of the phenylenediamine aromatic ring and C=C [35]. In addition, at the crest of 1612 cm^−1^, the strength of the absorption peak increases with the increase of HCl concentration, which is ascribed to the chemical changes in the PANI polymer chain induced by hydrogen ions making the stretching vibration of the Raman active C=C ring become more prominent.

**Figure 4:**
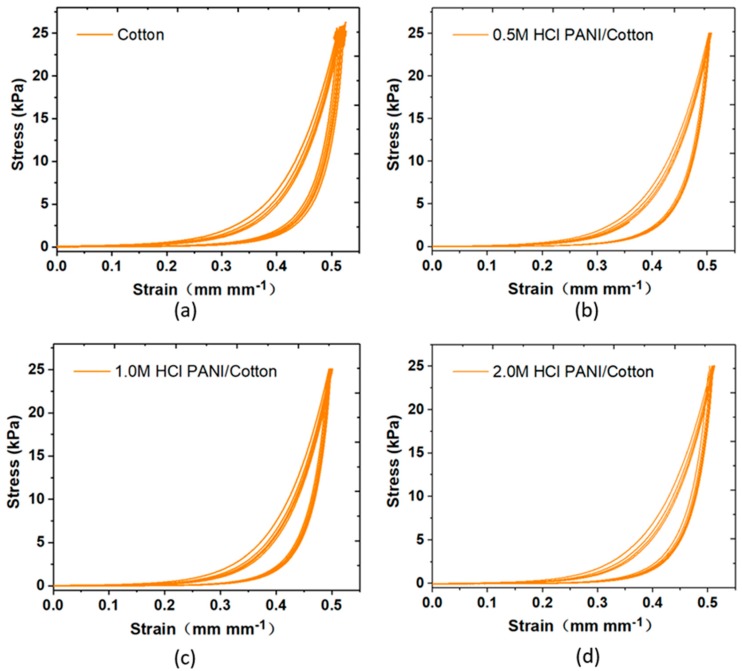
Consecutive compression tests on the PANI-coated nonwoven fabric. (**a**) Cotton, (**b**) 0.5 M HCl PANI/cotton, (**c**) 1.0 M HCl PANI/cotton, and (**d**) 2.0 M HCL PANI/cotton.

Conductivity analysis was performed to figure out the effect of HCl concentration on electrical conductivity. HCl is a strong acid that can effectively provide H^+^ for the medium as PANI dopant to increase the conductivity of the PANI/cotton composite. Seen in Table S1, with HCl concentration increased from 0.5 mol/L to 2.0 mol/L, the conductivity of PANI/Cotton increased by about two orders of magnitude reaching 0.97 ± 0.25 kΩ/sq. To evaluate the mechanical properties of PANI modified fabrics, we tested the strain response function (as shown in Figure 4). Figure 4a shows an unmodified pure cotton fabric with a deformation of 52% at 25 kPa loading. When the PANI fabric doped with HCl of different concentrations is subjected to the pressure of 25 kPa, the deformation is also as high as 50%, which is almost the same as the fabric without modification. This indicates that the modified conductive fabric is also soft and prone to deformation. Effective modulus of elasticity (E_eff_) is defined as the slope of the function curve of pressure-strain. It can be seen from the figure that the conductive fabric has a very small E_eff_ under low pressure, rising with increasing pressure. At 5 kPa loading, E_eff_ of the unmodified cotton fabric is 73.6 kPa. While after modification, E_eff_ of the conductive fabric doped with three different concentration of HCl is 75.6 kPa, 77.1 kPa and 76.3 kPa, respectively. This further indicates that the mechanical properties remain almost unchanged before and after modification. In addition, for previously reported flexible detectors which commonly used PDMS and hydrogel substrates, the E_eff_ is usually greater than 200 kPa [36], which is far more than the effective elastic modulus of PANI decorated conductive fabric. This may be due to the existing air gaps between fibers which result in compressed conductive fabric with a lower elastic modulus.

### 3.2. Electromechanical Characteristics of the Flexible Pressure Sensor

**Figure 5:**
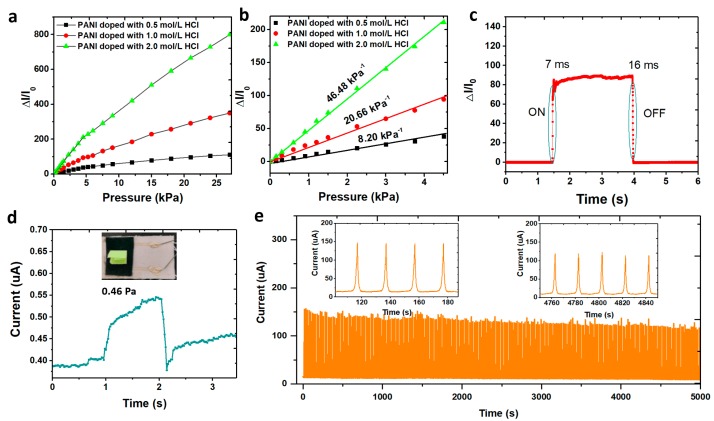
Evaluation of electromechanical performances. (**a**) Relative current change–pressure relationship of three pressure sensors with different concentrations of HCl; (**b**) Magnified curves from 0 to 4.5 kPa, showing its superior linearity over a large detection range; (**c**) Response/release time of the device; (**d**) Current response to the loading and removal of a small piece of paper on the PANI-based pressure sensor, corresponding to a pressure of only 0.46 Pa; (**e**) The cycling test of the sensor under 5 kPa pressure.

The sensitivity is an essential index to measure the performance of the pressure sensor. The graph in Figure 5a represents the function curve of the current change with the change of pressure where the slope represents the device sensitivity. It can be seen that the sensitivity will be increased with the concentration of HCl and the conductivity of the conductive fabric. The PANI coated fabric doped with 2.0 mol/L HCl has a higher conductivity than the others and the device constructed has the highest detection sensitivity. The resulting pressure sensors maintained ultra-sensitivity of 46.48 kPa^−1^ in a wide linear range (<4.5 kPa), which is among the best results for wearable pressure sensors (Table S2). In addition, linearity is also an important performance index of the detector. If the pressure sensors have a narrow linearity, the current signal will be distorted and it will not correctly reflect the applied load. As shown in Figure 5b, our PANI based pressure sensor has a superior linearity over a large detection range (0–4.5 kPa). This is mainly attributed to the fact that the PANI based pressure sensor utilizes non-woven fabrics with random fiber distribution, of which its surface highly conforms to the Gaussian distribution. The initial contact point of the randomly distributed microstructure will saturate the contact area under a certain pressure and new contact points will increase sharply to compensate the whole resistance change so that the current response can remain linear under a large pressure range. Figure 5c and internal illustrations show the response time of the resistive flexible pressure sensor during rapid loading and unloading. When there is no pressure applied to the device, the current does not change. With the pressure of 2 kPa applied, the current of the resistive flexible sensor increases by 80 times within 7 ms. Subsequently, the applied pressure was rapidly removed and the current was fleetly restored to the initial state within 16 ms. The above experimental results show that our PANI based pressure sensor has excellent response characteristics during loading and unloading. In addition, in order to further explore the minimum resolution of the device, we used a small piece of paper as the source of pressure to test the performance on pressure response of the pressure sensor (see Figure 5d). When a small piece of paper was placed on the surface of the device which was equivalent to applying pressure of only 0.46 Pa, the current passing through the device rapidly increased from 0.40 μA to 0.49 μA, improved by 22.5%, indicating that the device could well distinguish ultra-low pressure. This is due to the fact that PANI conductive fabric has numerous fabric fibers at micron level on its surface, which can be deformed under small pressure. Therefore, the contact area between the conductive fabric and the bottom electrode was increased, which further increased the flow current. In addition, a 5 kPa pulse pressure was repeatedly applied for 5000 s (loading frequency: 0.05 Hz) on the sensing surface and the current changes of the device were recorded in real time to detect its stability and durability. As shown in Figure 5e, when the pressure was loaded on the flexible pressure sensor, the current rapidly increased to 150 μA, which restored to the original state after unloading. During the stability test for 250 cycles, the current response of the pressure sensor was nearly the same. These results show that the flexible pressure sensor has high response stability and high reliability to repeat pulse pressure detection.

### 3.3. Real-Time Detection of Physiological Signals

**Figure 6:**
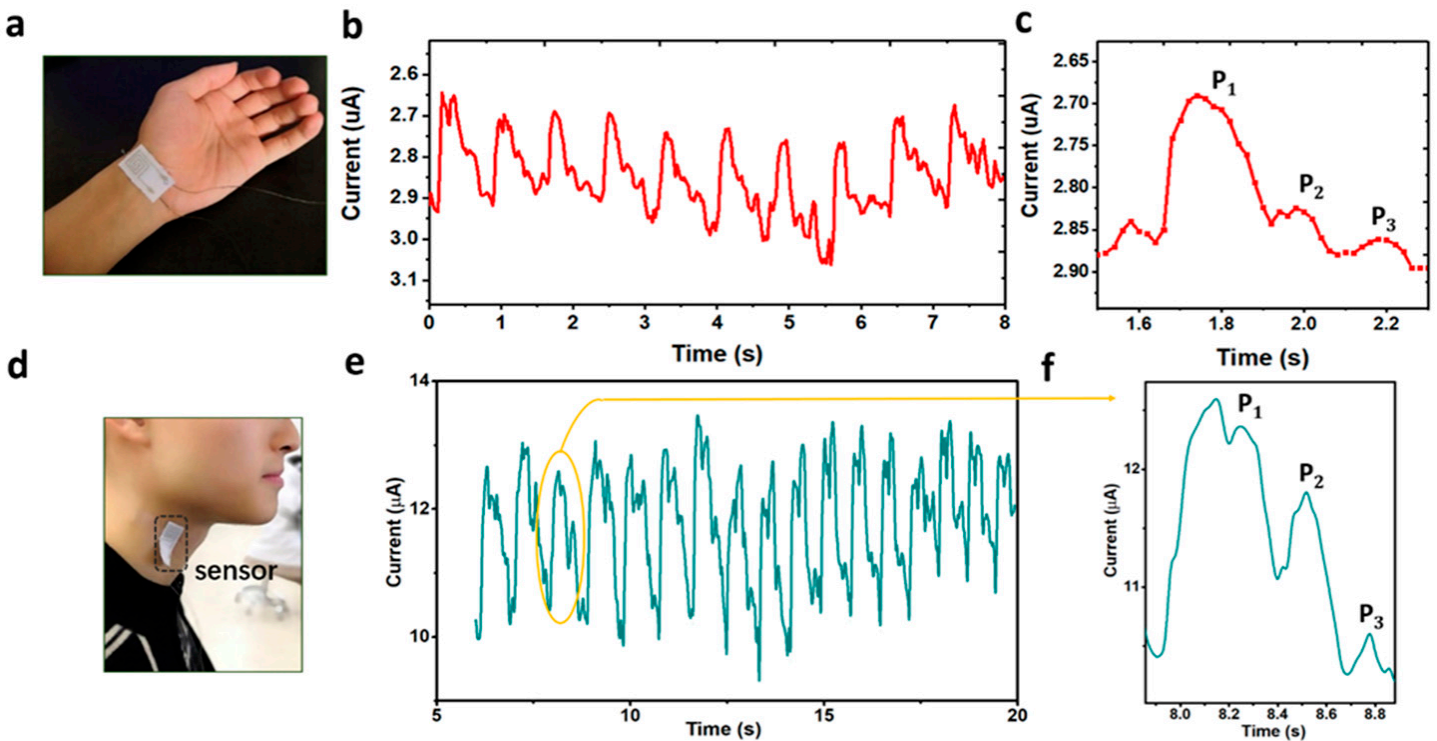
Real-time detection of different physiological signals by using the PANI-based flexible pressure sensor. (**a**) Photograph of a pressure sensor attached on the wrist for wrist pulses detection; (**b**,**c**) Wrist pulse waveform of the test pressure sensor and one single-pulse waveform; (**d**) Optical image of our textile sensor attached on the neck for arterial pulse waves detection (**e**,**f**) Neck pulse waveform of the test pressure sensor and one single-pulse waveform.

Because of its flexibility, high sensitivity and linearity, the PANI based pressure sensor would be suited as a part of the wearable applications. Typically, arterial pulses from radial artery and carotid artery could be detected by the sensors (attached on the wrist and neck, respectively, using an adhesive bandage), directly acquiring the pulse wave in a noninvasive manner. Thereout, significant information about the physiological condition of human arteries could be collected and provided for health monitoring. Figure 6a shows that the prepared pressure sensor accurately fitted the wrist over the radial artery. The corresponding pulse wave was shown in Figure 6b and its enlarged view of a single pulse cycle is shown in Figure 6c, from which a typical artery pulse wave composed of three distinguishable wave crests including percussion wave (P_1_), tidal wave (P_2_) and dicrotic wave (P_3_), were observed. Subsequently, Figure 6d and Figure 4e demonstrate the real-time detection of the waveform signals derived from the carotid artery when the flexible sensor was mounted on the human neck. Similarly, three clearly distinguishable component waves (P_1_, P_2_ and P_3_, respectively) were observed (shown in Figure 6f). In the assessment of the physical condition of the cardiovascular system, the radial augmentation index, defined as P_2_/P_1,_, is valuable information for diagnosing increased arterial stiffness (a marker of subclinical atherosclerosis and higher cardiovascular risk) [37].

### 3.4. Spatial Mapping for Pressure Distribution

**Figure 7:**
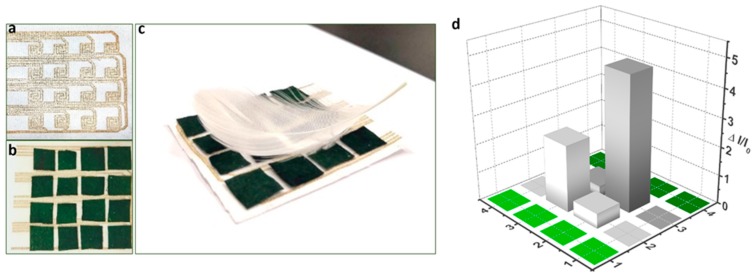
Pressure mapping by the PANI-based pressure sensing arrays. (**a**) a screen-printed sensor array with 4 × 4 pixels; (**b**) A photograph of the sensing array; (**c**) The sensing array pressed with a feather; (**d**) 3D bar graph showing the real-time relative current change.

To test the feasibility of applying the PANI based pressure sensor to wearable electronics and artificial electronic skin, the detection of spatial pressure distribution is a crucial part. Herein, we designed a sensor pixel sensing array (see Figure 7a) to monitor the change of pressure distribution. As shown in Figure 7b, the 4 × 4 sensor pixels sensing matrix (each is about 5 × 5 mm^2^) on textile substrate prepared by screen-printing of silver paste was constructed and each sensor pixel was secured with VHB film tape. When a piece of feather was placed on the sensor array (Figure 7c), the output current intensity was illustrated literally by the pixel bars which represent the pressing area in a 3D bar chart, as shown in Figure 7d.

## 4. Conclusions

In summary, PANI-coated fabric was used as the active material to construct high performance, flexible, all fabric pressure sensors with a bottom interdigitated textile electrode. Due to the unique hierarchical structures and large surface roughness of the PANI coated fabric, the resulting pressure sensors maintained ultra-sensitivity of 46.48 kPa^−1^ in a wide linear range (<4.5 kPa), which is among the best results for wearable pressure sensors. Moreover, the all fabric pressure sensors were able to achieve fast response/relaxation time (7/16 ms), low detection limit (0.46 Pa) and high stability (>250 loading/unloading cycles). Benefitting from these remarkable electromechanical properties, our sensors were able to detect physiological signals, such as wrist pulse and carotid pulse. Moreover, we demonstrated its excellent performance in monitoring spatial pressure distribution as the pixelated array. With its low cost and high performance, the sensor is promising for practical applications in wearable electronics and human health monitoring.

## Supplementary Materials

The following are available online at https://www.mdpi.com/2073-4360/11/7/1120/s1, Table S1: Sheet resistance of the polyaniline (PANI) coated fabric, Table S2: Comparison of reported flexible pressure sensors.
