# Supplementary material for: Polyaniline Nanofiber Wrapped Fabric for High Performance Flexible Pressure Sensors
Source: Polymers (Basel). 2019 Jul 2;11(7):1120. doi: 10.3390/polym11071120 (PMC6680572; doi:10.3390/polym11071120)
Supplement: Supplementary file 1 [file polymers-11-01120-s001.pdf]

Supporting Information:

Polyaniline Nanofiber Wrapped Fabric for High Performance Flexible Pressure Sensors

Kangning Liu,[a,b] Ziqiang Zhou,[a,b] Xingwu Yan,[a] Xiang Meng,[a] Hua Tang ,[a] Konggang Qu,[c] Yuanyuan Gao,[a] Ying Li,[a*] , Junsheng Yu[b] and Lu Li[a*]

[a]Research Institute for New Materials Technology, Chongqing University of arts and sciences, Chongqing 402160, P. R. China

[b]State Key Laboratory of Electronic Thin Films and Integrated Devices, School of Optoelectronic Science and Engineering, University of Electronic Science and Technology of China (UESTC), Chengdu 610054, P. R. China

[c]School of Chemistry and Chemical Engineering, Liaocheng University, Liaocheng, 252059, China

Table S1. Sheet resistance of the polyaniline (PANI) coated fabric

Test samples	Concentration of HCl	Sheet resistance (kΩ/sq)
Polyaniline fabric	0.5 mol/L	110.85 ± 16.20
Polyaniline fabric	1.0 mol/L	32.90 ± 8.81
Polyaniline fabric	2.0 mol/L	0.97 ± 0.25

Table S2. Comparison of reported flexible pressure sensors.

**Device type**	**Active materials**	**Linear pressure range**	**Sensitivity**	**Response time (ms)**	**Ref.**
Resistive	CNT	<1.5 kPa	14.4 kPa^{-1} below 3.5 kPa, 7.8 kPa^{-1} for 3.5-15 kPa	24	1
Resistive	Graphene	0 to 10 kPa	8.5 kPa^{-1}	40	2
Resistive	ACNT/ Graphene	0 to 0.3 kPa	19.8 kPa^{-1} below 0.3 kPa, 0.27 kPa^{-1} for 0.3-5.8 kPa	<16.7	3
Resistive	CSilkNM	<0.5 kPa	34.47 kPa^{-1} for 0.8-400 Pa, 1.16 kPa^{-1} for 400-5000 Pa	<16.6	4
Resistive	Graphene	0 to 2.6 kPa	25.1 kPa^{-1}	120	5
Resistive	Graphene	0 to 25 kPa	1.2 kPa^{-1}	-	6
Resistive	Graphene	0 to 0.2 kPa	110 kPa^{-1}	30	7
Resistive	CB/PVDF-decorated knitted fabric	<1.2 kPa	≈1.5 kPa^{-1}	2	8
Resistive	SWCNT film	0.6 Pa to 300 kPa	1.8 kPa^{-1}	<10	9
Resistive	Carbonized crepe paper	0 to 0.42 kPa	5.67 kPa^{-1}	<30	10
Resistive	Laser-scribed graphene	0 to 50 kPa	0.96 kPa^{-1} for 0-50 kPa, 0.005 kPa^{-1} for 50-113 kPa	72	11
Capacitance	CNT fiber/Ecoflex	0.38 Pa to 0.05 kPa	0.034-0.05 kPa^{-1} below 0.1 kPa, 0.5 MPa^{-1} above 10 kPa	≈63	12
Resistive	PANI	0 to 1.6 kPa	<1 kPa^{-1}	400	13
Resistive	PANI	<2.2 kPa	Maximum 0.89 kPa^{-1}	-	14
Resistive	PANI	0 to 4.5 kPa	46.48 kPa^{-1}	7	This work

CNT: carbon nanotube. ACNT: aligned carbon nanotube. CSilkNM: carbonized silk nanofiber membranes SWCNT: single-walled carbon nanotube. PVDF: polyvinylidene fluoride.

References:

1. Liu, M.; Pu, X.; Jiang, C.; Liu, T.; Huang, X.; Chen, L.; Du, C.; Sun, J.; Hu, W.; Wang, Z. L. Large-Area All-Textile Pressure Sensors for Monitoring Human Motion and Physiological Signals. *Adv. Mater.* **2017,** 29, 1703700.
2. Bae, G. Y.; Pak, S. W.; Kim, D.; Lee, G.; Kim, D. H.; Chung, Y.; Cho, K. Linearly and Highly Pressure-Sensitive Electronic Skin Based on a Bioinspired Hierarchical Structural Array. *Adv. Mater.* **2016,** 28, 5300-5306.
3. Jian, M.; Xia, K.; Wang, Q.; Yin, Z.; Wang, H.; Wang, C.; Xie, H.; Zhang, M.; Zhang, Y. Flexible and highly sensitive pressure sensors based on bionic hierarchical structures. *Adv. Funct. Mater.* **2017,** 27, 1606066.
4. Wang, Q.; Jian, M.; Wang, C.; Zhang, Y. Carbonized Silk Nanofiber Membrane for Transparent and Sensitive Electronic Skin. *Adv. Funct. Mater.* **2017,** 27, 1605657.
5. Pang, Y.; Zhang, K.; Yang, Z.; Jiang, S.; Ju, Z.; Li, Y.; Wang, X.; Wang, D.; Jian, M.; Zhang, Y.; Liang, R.; Tian, H.; Yang, Y.; Ren, T.-L. Epidermis Microstructure Inspired Graphene Pressure Sensor with Random Distributed Spinosum for High Sensitivity and Large. *ACS Nano.* **2018,** 12, 2346-2354.
6. Shi, J.; Wang, L.; Dai, Z.; Zhao, L.; Du, M.; Li, H.; Fang, Y. Multiscale Hierarchical Design of a Flexible Piezoresistive Pressure Sensor with High Sensitivity and Wide Linearity Range. *Small.* **2018,** 14, 1800819.
7. Xia, K.; Wang, C.; Jian, M.; Wang, Q.; Zhang, Y. CVD growth of fingerprint-like patterned 3D graphene film for an ultrasensitive pressure sensor. *Nano. Res.,* **2018**, 11, 1124-1134.
8. Luo, N.; Zhang, J.; Ding, X.; Zhou, Z.; Zhang, Q.; Zhang, Y. -T.; Chen, S. -C.; Hu, J. -L.; Zhao, N. Textile-Enabled Highly Reproducible Flexible Pressure Sensors for Cardiovascular Monitoring. *Adv. Mater. Technol.* **2017,** 3, 1700222.
9. Wang, X.; Gu, Y.; Xiong, Z.; Cui, Z.; Zhang, T. Silk-Molded Flexible, Ultrasensitive, and Highly Stable Electronic Skin for Monitoring Human Physiological Signals. *Adv. Mater.* **2014,** 26, 1336-1342.
10. Chen, S.; Song, Y.; Xu, F. Flexible and Highly Sensitive Resistive Pressure Sensor Based on Carbonized Crepe Paper with Corrugated Structure. *ACS Appl. Mater. Interfaces,* **2018**, 10 , 34646-34654.
11. Tian, H.; Shu, Y.; Wang, X. F.; Mohammad, M. A.; Bie, Z.; Xie, Q. Y.; Li, C.; Mi, W. T.; Yang, Y.; Ren, T. L. A Graphene-Based Resistive Pressure Sensor with Record-High Sensitivity in a Wide Pressure Range. *Sci. Rep*., **2015**, 5, 8603.
12. Park, S.; Kim, H.; Vosgueritchian, M.; Cheon, S.; Kim, H.; Koo, J. H.; Kim, T. R.; Lee, S.; Schwartz, G.; Chang, H.; Bao, Z. Stretchable Energy-Harvesting Tactile Electronic Skin Capable of Differentiating Multiple Mechanical Stimuli Modes. *Adv. Mater.*, **2014**, 26, 7324.
13. Tang, X.; Wu, C.; Zhang, T.; Zhou, T.; Wang, H.; Xie, C.; Zeng, D. A Low-Cost

Polyaniline@Textile-Based Multifunctional Sensor for Simultaneously Detecting Tactile and Olfactory Stimuli. *Macromol. Mater. Eng.*, **2018**, 1800340.

14. Liu, H.; Wu, Y.; Chen, S.; Liu, S.; Wang, P.; Xue, F.; Liu, L. A flexible and multifunctional electronic nose using polyaniline/cotton fibrous membrane with a hierarchical structure. *J.Matlet.*, **2018**, 09, 046.
